# *MUC1* gene polymorphism rs4072037 and susceptibility to gastric cancer: a meta-analysis

**DOI:** 10.1186/2193-1801-3-599

**Published:** 2014-10-13

**Authors:** Xinyang Liu, Zhichao Wang, Xiaowei Zhang, Jinjia Chang, Wenbo Tang, Lu Gan, Zheng Wu, Jin Li

**Affiliations:** Department of Medical Oncology, Fudan University Shanghai Cancer Center; Department of Oncology, Shanghai Medical College, Fudan University, Shanghai, 200032 PR China; Liver Cancer Institute, Zhongshan Hospital, Fudan University, Shanghai, 200032 China

**Keywords:** MUC1, Polymorphism, Genetic, Stomach neoplasms, Meta-analysis

## Abstract

The association between *MUC1* polymorphism rs4072037 and the risk of gastric cancer has been described in several studies. However, these studies yielded inconsistent results, especially in different pathological type of gastric cancer. Therefore, we performed this meta-analysis to evaluate the relationship between *MUC1* gene polymorphism and gastric cancer susceptibility. A comprehensive database search was performed to identify eligible studies. Odds ratios with 95% confidence intervals were calculated to assess the strength of the association between *MUC1* rs4072037 and risk of gastric cancer. Subgroup analyses, publication bias, and sensitivity analyses were also conducted. PubMed, EMBASE, Web of Science and CNKI databases were systematically searched to identify relevant studies. A total of 9 studies (12 datasets) were included in the meta-analysis including 10,410 cases and 11,437 controls. Overall, the G allele at rs4072037 of *MUC1* gene was associated with a significant decreased gastric cancer risk (OR=0.70, 95% CI: 0.64–0.76). The association was significant in both anatomic location and pathological subtype subgroup analyses. However, the association was detected in Asian rather than Caucasian. Our findings demonstrate that the presence of the G allele at rs4072037 of the *MUC1* gene may contribute to protection against gastric cancer in Asian. Further large studies of multiethnic groups are needed to validate these findings.

**Electronic supplementary material:**

The online version of this article (doi:10.1186/2193-1801-3-599) contains supplementary material, which is available to authorized users.

## Introduction

Gastric cancer (GC) is the fourth most common cancer and the second leading course of cancer-related deaths worldwide, with an estimated 989,600 new cases and 738,000 deaths in 2008 (Jemal et al. 2011). Although the incidence and mortality of GC have decreased over the past few decades, it is still a heavy burden in developing areas like East Asia, Eastern Europe, and South America. The etiology of GC remains unclear, although environmental risk factors and host genetic factors are both thought to play a role in the GC carcinogenesis (Wu et al. 2005). *Helicobacter pylori* is the single most important etiological agent in the pathogenesis of GC (Blaser 2000), and it is becoming increasingly clear that several specific host genes are involved in response to *H.pylori* colonization, immune escape and mucosal injury in the stomach.

*MUC1* gene is a member of the mucin family. It encodes a membrane-bound glycoprotein, which functions in protection of epithelial surfaces against environmental insults (Gendler 2001). For example, it can block the adhesion of *H.pylori* blood group antigen-binding adhesin (BabA) and sialic acid-binding adhesin (SabA) to the gastric mucosa, thus limiting *H.pylori* colonization (Linden et al. 2009; Skoog et al. 2012). The association between *MUC1* polymorphism rs4072037 and the risk of gastric cancer has been described in previous studies (Xu et al. 2009; Jia et al. 2010) with a candidate gene approach. Recently, a genome-wide association study (GWAS) (Abnet et al. 2010) performed in Chinese population identified the same suspicious locus in the scanning phase but not in the second phase. After combining the two phases, it still failed to reach genome-wide significance. Similarly, case–control study in Japan demonstrated that rs4072037 was only associated with diffuse-type gastric cancer but not intestinal-type gastric cancer. Since then, several studies were performed to validate these findings. Although the majority of the results are similar, inconsistency exists regarding to the role of rs4072037 in different pathological types of gastric cancer and different ethnic groups. A meta-analysis has been conducted by Zheng et al. (Zheng et al. 2013). However, their meta-analysis has some obvious limitations. Therefore, we performed this meta-analysis to evaluate the relationship between *MUC1* gene polymorphism at rs4072037 and gastric cancer susceptibility and assess the effect size of the association in order to clarify the inconsistency among published studies.

## Materials and methods

### Literature search strategy

We performed a systematic review and meta-analysis on *MUC1* gene polymorphisms and gastric cancer susceptibility in accordance with the PRISMA Statement (Preferred Reporting Items for Systematic Reviews and Meta-Analyses) (Liberati et al. 2009) (Additional file 1). We searched MEDLINE (PubMed), EMBASE, Web of Science, and CNKI (China National Knowledge Infrastructure) without language restrictions to December 12, 2013, using the following search algorithms: ("MUC1" OR "mucin1" OR "1q22") AND ("polymorphism" OR "SNP") AND ("gastric neoplasms" OR "gastric cancer" OR "gastric carcinoma"). In addition, reference lists of identified studies and related GWAS were also hand-searched to identify potential eligible studies.

### Selection criteria

Study eligibility was determined independently by two reviewers (LXY and ZXW). Disagreements were solved by consensus. Studies were considered for inclusion if they meet the following criteria: (i) evaluated *MUC1* gene polymorphism rs4072037 and gastric cancer susceptibility, (ii) were cohort-based or case–control studies, and (iii) reported data necessary to calculate the odds ratios (ORs) with corresponding 95% confidence intervals (95% CIs). If such data were unavailable, attempts were made to contact the first author and/or corresponding author via e-mail to provide the missing data before the study was excluded from the final analysis. The major exclusion criteria included: (i) reviews, case-only studies, or familial studies, (ii) lack of sufficient data for calculation of ORs with 95% CIs, and (iii) duplication of previous publications or replicated samples.

### Data extraction and quality assessment

Data extraction was carried out independently by two reviewers (LXY and ZXW) using a pre-defined standard form. Disagreements were resolved by discussion with a third author (WZC). From each study, the following information was extracted: first author’s name, year of publication, ethnicity of the patients, source of control groups (population- or hospital-based controls), age, sex, genotyping method, Hardy-Weinberg equilibrium (HWE) of controls, and frequency of various genotypes in cases and controls, adjusted ORs and 95% CIs. If allele frequencies were not given, they were calculated from the corresponding genotype distributions. If genotype frequencies were not given, they were calculated from allele frequencies only when the study was in accordance with HWE. For one study (Saeki et al. 2011), which included datasets of different ethnic populations and reported the results separately, the data were collected separately and the datasets were recognized as independent studies. Study quality was assessed independently by LXY and ZXW using the 10-point scoring scale for quality of genetic association studies proposed by Clark and Baudouin (Clark and Baudouin 2006).

### Statistical analysis

ORs and 95% CIs were used to assess the strength of the associations between *MUC1* polymorphism rs4074037 and gastric cancer risk. OR represents the odds that a certain genotype occurring in gastric cancer patients, compared to the odds of the genotype occurring in controls. A *P* value of< 0.05 in a Z-test indicated statistical significance for the associations. Adjusted ORs and 95%CIs were used if reported. Otherwise, the pooled ORs and 95% CIs without adjustments were calculated for the following genotypic models: allele (G vs. A), homozygote (GG vs. AA), heterozygote (AG vs. AA), dominant (GG/AG vs. AA), and recessive (GG vs. AG/AA). If adjusted ORs and 95% CIs were reported based on A allele vs. G allele, they were converted to G allele vs. A allele.

A Cochrane chi-square-based Q-test was performed to test the heterogeneity among studies or cohorts. The *I*^*2*^ tests were performed to assess the statistical heterogeneity, and the Q-statistic tests with *P*< 0.10 were used to define a significant degree of heterogeneity. A fixed-effect model was used when there was no heterogeneity (*P* ≥0.10 for Q-test) (Mantel and Haenszel 1959), otherwise a random-effect model was used (DerSimonian and Laird 1986). We also estimated the statistical power of each individual study as determined by the probability of detecting a true association between MUC1 polymorphism rs4072037 and gastric cancer risk at the 0.05 level of significance. The estimation was based on the method described earlier (Schlesselman 1982).

For exploration of heterogeneity, subgroup analyses were performed based on anatomic location, pathological subtype, ethnicity, source of control, sample size (≤1000 and >1000 subjects), quality scores (score >7 or ≤7) adjustment and statistical power. Sensitivity analyses were performed by deletion of a single study each time to reflect the influence of the individual data set on the pooled ORs. Begg’s funnel plots (Egger et al. 1997) were used to assess the publication bias. Statistical analyses were conducted using Review manager Version 5.1 (Copenhagen, The Nordic Cochrane Centre, The Cochrane Collaboration, 2011).

## Results

### Characteristics of the studies

**Figure 1:**
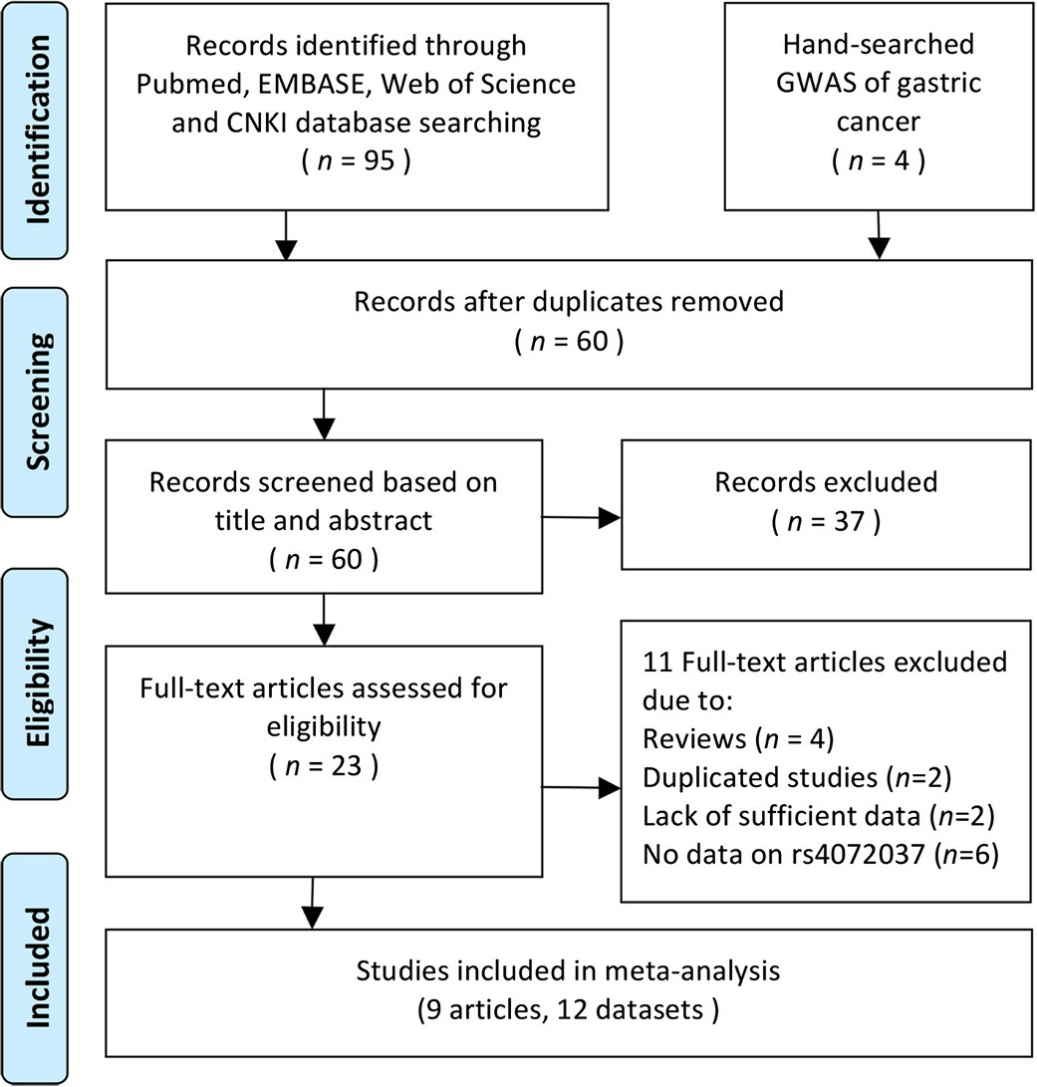
**Flow diagram of the study process.** CNKI, China National Knowledge Infrastructure; GWAS, genome-wide association studies.

The process of selection of studies in the meta-analysis is summarized in a flow diagram (Figure 1). Database search revealed 99 potentially relevant publications. Eventually, 9 studies (Xu et al. 2009; Jia et al. 2010; Abnet et al. 2010; Yang et al. 2012; Palmer et al. 2012; Zhang et al. 2011; Saeki et al. 2011; Li et al. 2013; Song et al. 2013) that consisted of 12 datasets were eligible based on the inclusion/exclusion criteria. The main characteristics of included studies are shown in a table (Additional file 2).

### Overall effects for meta-analysis

All the 12 datasets including 10,410 cases and 11,437 controls presented enough data on the allele model. Two studies did not report the frequencies of each genotype and HWE. Of them, one study (Saeki et al. 2011) (including 4 datasets) only reported adjusted ORs and corresponding 95% CIs under the allele model, and the other study (Abnet et al. 2010) reported adjusted ORs and corresponding 95% CIs under allele, homozygote and heterozygote model. Thus, they were not included in the meta-analyses under some of the models.

**Figure 2:**
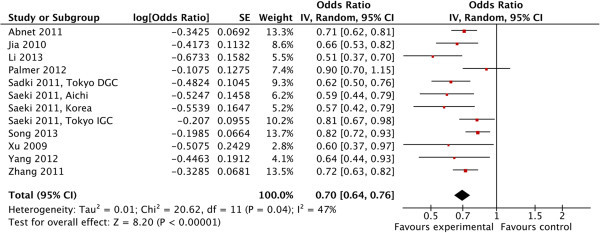
**Forest plot describing** ***MUC1*** **rs 4072037 and susceptibility to gastric cancer under the allele model.** The horizontal lines represent 95% CIs for estimating the outcome of the G allele versus the A allele in the meta-analysis. (■) Overall estimates of the effects. CI, confidence interval; OR, odds ratio.

In the overall analysis, we detected a significant association between the G allele at rs4072037 with decreased gastric cancer risk under the allele model (G vs. A; OR=0.70, 95% CI: 0.64–0.76; 10,410 cases and 11,437 controls.) (Table 1, Figure 2), homozygote model (GG vs. AA; OR= 0.63, 95% CI: 0.52–0.76; 8,843 cases and 8,341 controls), heterozygote model (AG vs. AA; OR=0.68, 95% CI: 0.63–0.73; 8,843 cases and 8,341 controls), dominant model (GG/AG vs. AA; OR=0.65, 95% CI: 0.54–0.77; 6,209 cases and 5,039 controls), and recessive model (GG vs. AG/AA; OR=0.76, 95% CI: 0.63–0.92; 6,209 cases and 5,039 controls). Significant heterogeneity was detected in allele and dominant models among studies.

**Table 1:** **Meta-analyses of the association between** ***MUC1*** **polymorphism rs4072037 and gastric cancer risk under alternative models**

Comparison	No. of datasets	Case	Control	OR (95% CI)	*P*for OR	*P*for heterogeneity	I ^2^(%)	Model
G vs. A	12	10410	11437	0.70 (0.64,0.76)	<0.00001	0.04	47	Random
GG vs. AA	8	8438	8341	0.63 (0.52, 0.76)	<0.00001	0.47	0	Fixed
AG vs. AA	8	8438	8341	0.68 (0.63,0 73)	<0.00001	0.28	19	Fixed
GG/AG vs. AA	7	6209	5039	0.65 (0.54, 0.77)	<0.00001	0.01	64	Random
GG vs. AG/AA	7	6209	5039	0.76 (0.63,0.92)	0.005	0.34	11	Fixed

OR, odds ratio; CI, confidence interval.

### Subgroup analysis

Subgroup analyses by anatomic location, pathological subtype, ethnicity, source of controls, sample size (≤1000 and >1000 subjects), quality score (score >7 or ≤7), adjustment and statistical power (≥0.8 or<0.8) were performed only under the allele model, which had the largest sample size among all genotypic models (Table 2). Subgroup analyses indicated that the G allele at rs4072037 was associated with decreased gastric cancer risk regardless of anatomic location and pathological subtype. The association was significant in cardia gastric cancer (OR=0.75, 95% CI: 0.62–0.91), non-cardia gastric cancer (OR=0.66, 95% CI: 0.58–0.74), diffuse-type gastric cancer (OR=0.65, 95% CI: 0.57–0.73) and intestinal-type gastric cancer (OR=0.75, 95% CI: 0.66–0.83) (Additional file 3). Similarly, stratification by ample size, quality score, statistical power and source of control did not alter the results. There was also no difference between adjusted and unadjusted results. Moreover, we have noticed that heterogeneity could be reduced significantly when stratified by pathological subtype, quality score and control source, and statistical power, which may partly explain the source of heterogeneity.

**Table 2:** **Subgroup meta-analyses of the association between** ***MUC1*** **polymorphism rs4072037 and gastric cancer risk under the allele model**

Comparison	No. of datasets	Case	Control	OR (95% CI)	*P*for OR	*P*for heterogeneity	I ^2^(%)	Model
**Overall**	12	10410	11437	0.70 (0.64,0.76)	<0.00001	0.04	47	Random
**Anatomic location**								
Cardia gastric cancer	5	2362	7426	0.75(0.62,0.91)	0.004	0.04	63	Random
Non-cardia gastric caner	5	5161	7426	0.66 (0.58,0.74)	<0.00001	0.15	43	Fixed
**Pathological subtype**								
Diffuse-type gastric cancer	6	1756	5191	0.65 (0.57,0.73)	<0.00001	0.29	19	Fixed
Intestinal-type gastric cancer	4	1727	3354	0.75 (0.66,0.83)	<0.00001	0.48	0	Fixed
**Ethnicity**								
Asian	10	9826	10853	0.69 (0.63,0.75)	<0.00001	0.05	47	Random
Caucasion	2	584	584	0.76 (0.56,1.04)	0.08	0.07	70	Random
**Sample size**								
>1000	6	8652	9614	0.73(0.69,0.78)	<0.00001	0.10	45	Fixed
≤1000	6	1758	1823	0.66 (0.59,0.75)	<0.00001	0.10	47	Fixed
**Quality score**								
>7	5	6183	5742	0.73 (0.65,0.82)	<0.00001	0.27	23	Fixed
≤7	7	4227	5695	0.69 (0.64,0.75)	<0.00001	0.22	28	Fixed
**Control source**								
Hospital-based	7	2683	3958	0.65 (0.59,0.72)	<0.00001	0.16	35	Fixed
Population-based	5	7727	7479	0.75 (0.70,0.81)	<0.00001	0.19	34	Fixed
**Adjustment**								
Yes	9	9688	10612	0.69 (0.63,0.76)	<0.00001	0.04	51	Random
No	3	722	825	0.74 (0.63,0.86)	0.0001	0.13	51	Fixed
**Statistical power**								
≥0.8	6	5595	8620	0.67(0.62, 0.72)	<0.00001	0.22	0.29	Fixed
<0.8	6	4815	4081	0.78(0.72,0.85)	<0.00001	0.27	0.22	Fixed

OR, odds ratio; CI, confidence interval.

Subgroup analyses were also performed according to ethnicity. The results indicated that G allele was associated with decreased gastric cancer risk in Asian (OR=0.69, 95% CI: 0.57–0.73) rather than Caucasian (OR=0.76, 95% CI: 0.56–1.04). Stratification of the Asian subgroup into Chinese, Japanese and Korean did not alter the results. Notably, the subgroup of Caucasian included fewer than three cohorts, which could not yield reliable results in the meta-analysis (Liberati et al. 2009).

### Sensitivity analysis

One cohort was excluded at each time to investigate the influence of the individual data set on the overall results. The statistical significance of the overall results was not altered when any single study was excluded, confirming the stability of the results. However, *P* for Q test could reduce significantly when excluding the study of Li et al. (Li et al. 2013) and Song et al. (Song et al. 2013).

### Publication bias

**Figure 3:**
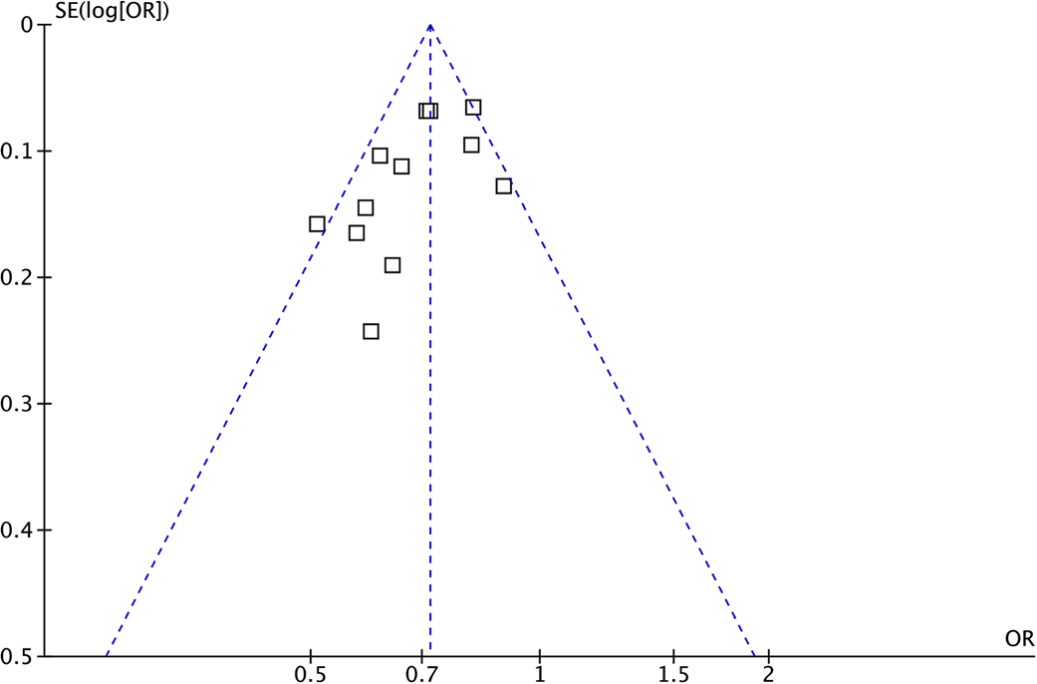
**Funnel plot of the association between** ***MUC1*** **rs4072037 and susceptibility to gastric cancer under the allele model.** OR, odds ratio; SE, standard error.

Begg’s funnel plots (Figure 3) were conducted to assess publication bias for reported comparisons of rs4072037 and association with gastric cancer. The funnel plots were all symmetrical, indicating that there was no publication bias in the studies of *MUC1* polymorphism and gastric cancer association (Begg’s test: *p*= 0.115, Egger’s test: *p*= 0.060).

## Discussion

The identification of genetic variants capable of modulating cancer development could be helpful for the early detection and design of targeted treatment and prevention strategies. With high interest in gene susceptibility to carcinogenesis, increasing efforts have been devoted to the study of genetic variants and gastric cancer risk. Since *MUC1* polymorphism has been reported to be associated with gastric cancer, several studies were performed to validate this finding. However, the results were inconsistent especially in different pathological type. Therefore, we performed the first meta-analysis to assess the relationship between *MUC1* gene polymorphism rs4072037 and gastric cancer susceptibility.

The meta-analysis included 10,410 gastric cancer patients and 11,437 controls, which was far larger than the sample size of the discovery study and the GWAS. Association of *MUC1* rs4072037 and gastric cancer susceptibility was detected under all genotypic models without significant heterogeneity, suggesting decreased gastric cancer risk for individuals carrying the G allele. Although some of the included studies did not have enough statistical power, this did not influence the overall results and confidence because in the subgroup analyses, studies with power larger than or less than 0.8 yielded similar results with statistical significance.

A large-sample-size Japanese study (Saeki et al. 2011) demonstrated that *MUC1* rs4072037 was associated with gastric cancer risk in diffuse-type gastric cancer rather than intestinal-type gastric cancer. However, our meta-analysis detected significant association in both subtypes. The sample size of the intestinal-type gastric cancer subgroup in our meta-analysis was twice larger than that in the Japanese study, indicating that our result was solid.

Subgroup analyses also revealed that G allele at rs4072037 was associated with gastric cancer risk in Asian rather than Caucasian. Interestingly, we notice that the allele frequencies of G allele were more than 0.5 in both the Caucasian studies, while G was the minor allele in all the Asian studies. This remarkable difference in the frequency of the G allele might be due to distinct genetic backgrounds of different ethnicities and this might attribute to the different susceptibility of G allele to gastric cancer. Further large studies of multiethnic groups are needed before a comprehensive conclusion could be made.

*MUC1* is a highly polymorphic transmembrane glycoprotein expressed on the surface of many epithelia, including gastric mucosa. It acts as a barrier against exogenous insults in normal epithelial cells. In contrast, once the cells lose cell polarity, *MUC1* protein interacts freely with other molecules including membrane receptors involved in cell growth and, consequently, promotes cell growth and acts for tumorigenesis (Kufe 2009). Interestingly, SNP rs4072037, located in exon 2 of *MUC1* gene, controls alternative splicing of the 5’-exon 2 region, resulting in both full-length transcripts and those lacking the polymorphic tandem repeat domain (Ng et al. 2008). The different protein products encoded by the two splice variants differ in the protective function of gastric mucosa (Saeki et al. 2011), which ultimately results in the difference in GC susceptibility. Therefore, our meta-analysis provided additional data supporting the potential functional role of *MUC1* in gastric cancer carcinogenesis, which needs to be authenticated through molecular and cellular approaches.

There is a recently published meta-analysis on *MUC1* rs4072037 and the risk of gastric cancer performed by Zheng et al. (Zheng et al. 2013) and we believe our study has some advantages over theirs’ for the following reasons. First, Zhang et al. (Zheng et al. 2013) falsely calculated the number of total controls in their manuscript. In one of the included studies conducted by Seaki et al. (Saeki et al. 2011), the "Diffuse, Tokyo data set" and "Intestinal, Tokyo data set" (referred to as data set "a" and "d" in Zheng et al’s manuscript) shared the same group of controls, thus the total number of controls should be 9,060 instead of 10,324 in the meta-analysis. Second, the breakdown of the patient groups as independent datasets in one study in the overall analyses may introduce analysis bias. This method should be used only if the combined data is not available. It is understandable that the study by Seaki et al. (Saeki et al. 2011) only reported the separate results in different ethnic groups instead of combined results, which is also regarded as independent datasets in our meta-analysis. However, the patients in the study by Abnet et al. (Abnet et al. 2010) should not be separated as combined data was also reported. Third, we have concluded that *MUC1* rs4072037 was associated with gastric cancer risk in Asian (OR=0.69, 95% CI: 0.63–0.75) rather than Caucasian (OR=0.76, 95% CI: 0.56–1.04), which is different from the conclusion of Zheng et al. (Zheng et al. 2013). We find that Zheng et al. (Zheng et al. 2013) may have drawn a wrong conclusion by adopting the fixed effect model with the *I*^*2*^ of 70% and *P* for Q-test of 0.07, while a random effect model should be used. Given these limitations and errors of the meta-analysis conducted by Zheng et al. (Zheng et al. 2013), their conclusions should be adopted with caution. Moreover, we have done a more profound literature search, and included three more studies (Yang et al. 2012) (Li et al. 2013) compared with the paper by Zheng et al. (Zheng et al. 2013) and our subgroup analyses were more detailed than that of Zheng et al. (Zheng et al. 2013). Besides all the parameters in Zheng et al’s paper (Zheng et al. 2013), we also included source of control, adjustment, sample size and quality in the subgroup analyses.

However, some limitations of our meta-analysis should be acknowledged when interpreting the results. First, we were unable to conduct stratified analyses based on possible confounders such as age, *H. pylori* infection, smoking status, and alcohol intake due to insufficient data. Second, the data for Caucasian was limited. Third, the breakdown of the patient groups as independent datasets in one study (Saeki et al. 2011) in the overall analyses may introduce analysis bias. However, this study only reported the separate results in different ethnic groups instead of combined results. Under this circumstance, although it is recommended that tests for funnel plot asymmetry should be used only when there are at least 10 studies included in the meta-analysis (Ioannidis and Trikalinos 2007), we still tested the publication bias because we have 12 datasets in 9 studies. In addition, it is not mentioned whether the cancer-free hospital-based controls were gastritis patients, which limits our analyses on the role of *MUC1* rs4072037 in gastric carcinogenesis when compared with the population-based healthy controls.

In conclusion, our meta-analysis summarizes the existing data on *MUC1* polymorphism rs4072037 and gastric cancer susceptibility. The results reveal that the presence of the G allele contributes to protection against gastric cancer in Asian, regardless of anatomic location and pathological subtype. Further large studies of multiethnic groups and investigation of confounders are warranted to validate these findings.

## Electronic supplementary material

Additional file 1: **PRISMA 2009 checklist.** (DOC 64 KB)

Additional file 2: **Characteristics of included studies.** (DOC 49 KB)

Additional file 3: **Forest plots describing subgroup analyses of** ***MUC1*** **rs4072037 and susceptibility to gastric cancer under the allele model.** (DOC 714 KB)
