# Supplementary material for: MUC1 gene polymorphism rs4072037 and susceptibility to gastric cancer: a meta-analysis
Source: Springerplus. 2014 Oct 13;3:599. doi: 10.1186/2193-1801-3-599 (PMC4198476; doi:10.1186/2193-1801-3-599)
Supplement: Supplementary file 2 — Additional file 2: Characteristics of included studies. (DOC 49 KB) [file 40064_2014_1298_MOESM2_ESM.doc]

**Additional file 2. Characteristics of the included studies.**

| **Study** | **Sample**  **Size** | **Source of Control** | **Anatomic Location** | **Pathological Subtype** | **Ethnicity** | **Genotyping method** | **Score** | **Age**  **(yr)** | **Male (%)** | **Adjustment** | **MAF** | **P_HWE_** | **Power** |
| --- | --- | --- | --- | --- | --- | --- | --- | --- | --- | --- | --- | --- | --- |
| Abnet 2010 | 2240/3302 | PB | CGC: 1213  NCGC: 917 |  | Chinese | Taqman | 8 | - | - | Age, sex, study | 0.125/0.159 | Not reported | 0.945 |
| Zhang 2011 | 1658/1883 | PB | CGC: 809  NCGC: 717 | IGC:1070  DGC:312 | Chinese | Taqman | 7 | 51.1/51.2 | 73.6/71.9 | Age,sex,smoking  ,drinking | 0.143/0.192 | Yes | 0.974 |
| Yang 2012 | 249/292 | HB  (cancer-free) |  |  | Chinese | Sequenom  MassARRAY | 8 | 54.8/58.7 | 71.5/59.9 | Age, sex | 0.157/0.207 | Yes | 0.320 |
| Palmer 2012 | 311/207 | PB | CGC: 122  NCGC: 184 | IGC:58  DGC:82 | US | PCR | 8 | - | - | Age, sex* | 0.534/0.560 | Yes | 0.089 |
| Xu 2009 | 138/241 | HB  (cancer-free) |  |  | Chinese | PCR-SSP | 8 | 57.6/56.6 | 66/66 | Age, sex* | 0.094/0.147 | Yes | 0.487 |
| Saeki 2011, Tokyo DGC | 606/1264 | HB  (cancer-free) |  | DGC | Japanese | High-density mapping | 7 | 54.7/64.7 | 52.8/67.2 | Age, sex,  risk allele number | 0.126/0.187 | Not reported | 0.923 |
| Saeki 2011, Tokyo IGC | 599/1264 | HB  (cancer-free) |  | IGC | Japanese | High-density mapping | 7 | -/64.7 | -/67.2 | Age, sex,  risk allele number | 0.157/0.187 | Not reported | 0.350 |
| Saeki 2011, Aichi | 304/1465 | HB  (cancer-free) |  | DGC | Japanese | High-density mapping | 7 | 57.0/59.2 | 65.5/74.9 | Age, sex,  risk allele number | 0.099/0.164 | Not reported | 0.852 |
| Saeki 2011, Korea | 452/372 | HB  (cancer-free) |  | DGC | Korean | High-density mapping | 7 | 53.4/50.7 | 51.3/57.5 | Age, sex,  risk allele number | 0.093/0.163 | Not reported | 0.853 |
| Jia 2010 | 273/377 | PB |  |  | Polish | SNPlex | 6 | - | 67.8/65 | Age, sex* | 0.427/0.531 | No | 0.746 |
| Li 2013 | 335/334 | HB  (cancer-free) | CGC:78;  NCGC:257 |  | Chinese | PCR | 7 | - | 68.7/67.1 | Age, sex, BMI, smoking and drinking status, and *H. pylori* serology | 0.11/0.20 | Yes | 0.897 |
| Song 2013 [ | 3245/1700 | PB  (Thyroid Disease Prevalence Study) | CGC:140;  NCGC:3086† | IGC:1932  DGC:781† | Korean | PCR | 8 | 60.5/52.1 | 67.9/48.3 | Age, sex | 0.11/0.13 | Yes | 0.644 |

A/B: case/control; SNP: single nucleotide polymorphism MAF: minor allele frequency; **P_HWE_**: P value of HWE in controls; PB: population based; HB: hospital based; CGC: cardia gastric cancer; NCGC: noncardia gastric cancer; †no data available on allele frequencies separated by cancer location or pathological type subgroups; *no adjusted ORs available under the allele model.
