# Supplementary material for: MUC1 gene polymorphism rs4072037 and susceptibility to gastric cancer: a meta-analysis
Source: Springerplus. 2014 Oct 13;3:599. doi: 10.1186/2193-1801-3-599 (PMC4198476; doi:10.1186/2193-1801-3-599)
Supplement: Supplementary file 3 — Additional file 3: Forest plots describing subgroup analyses of MUC1 rs4072037 and susceptibility to gastric cancer under the allele model. (DOC 714 KB) [file 40064_2014_1298_MOESM3_ESM.doc]

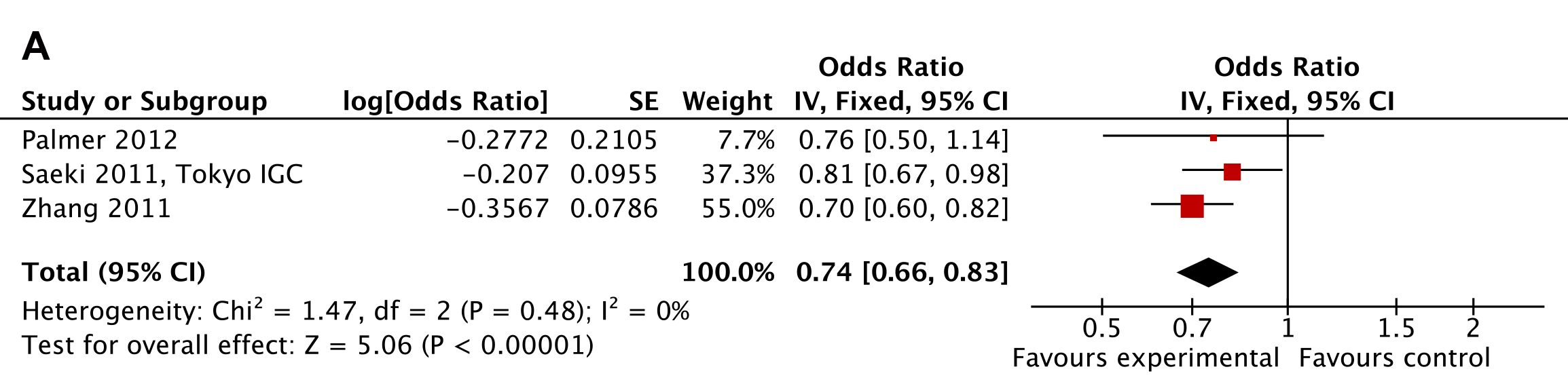


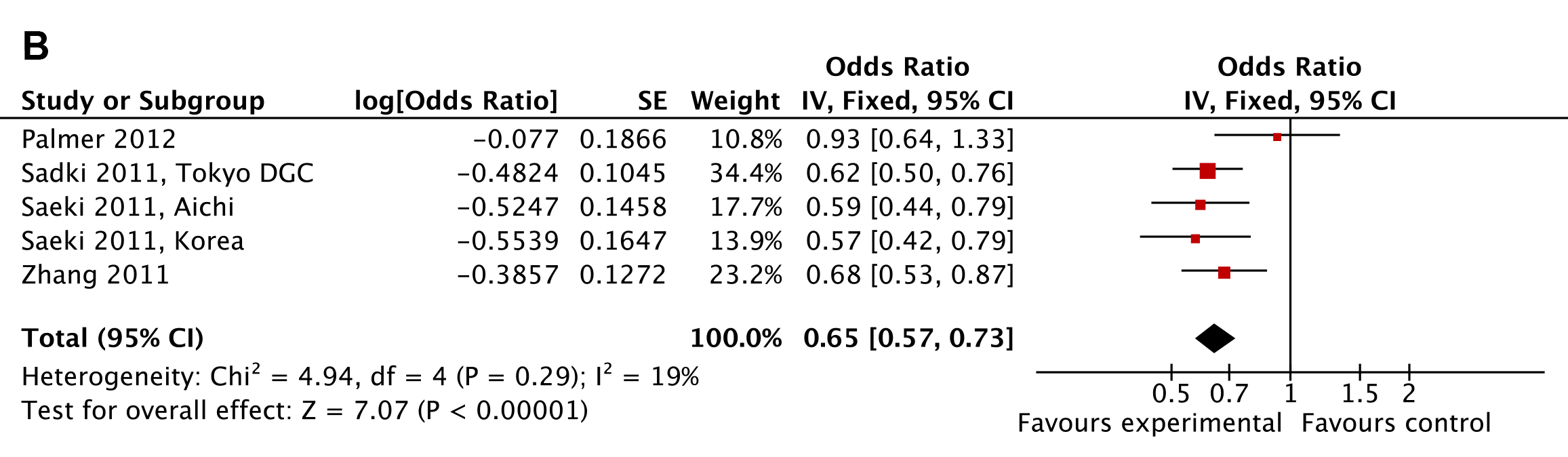


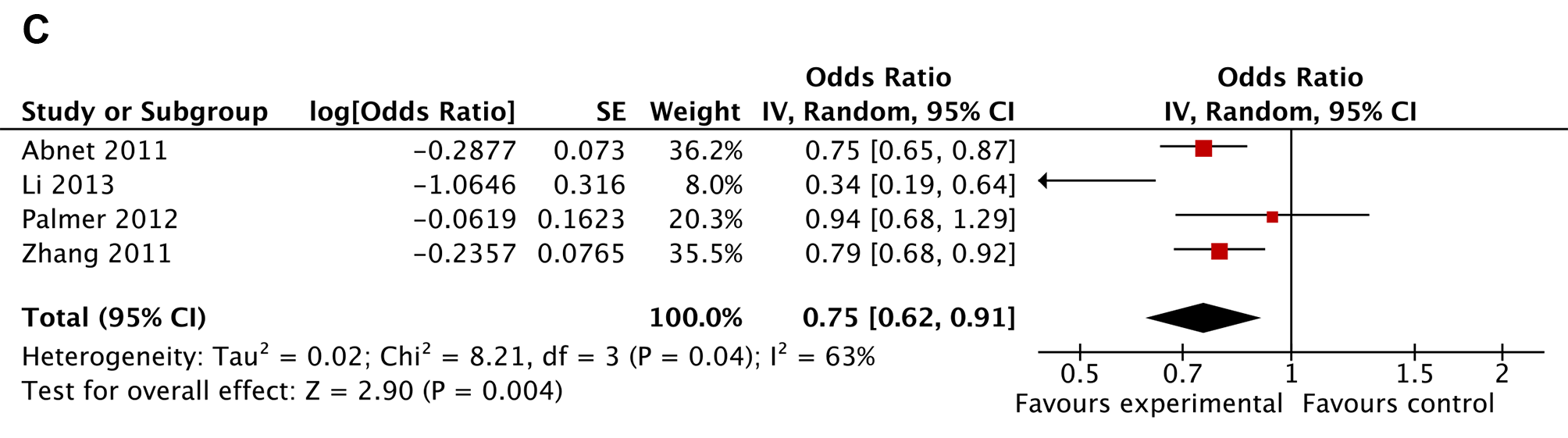


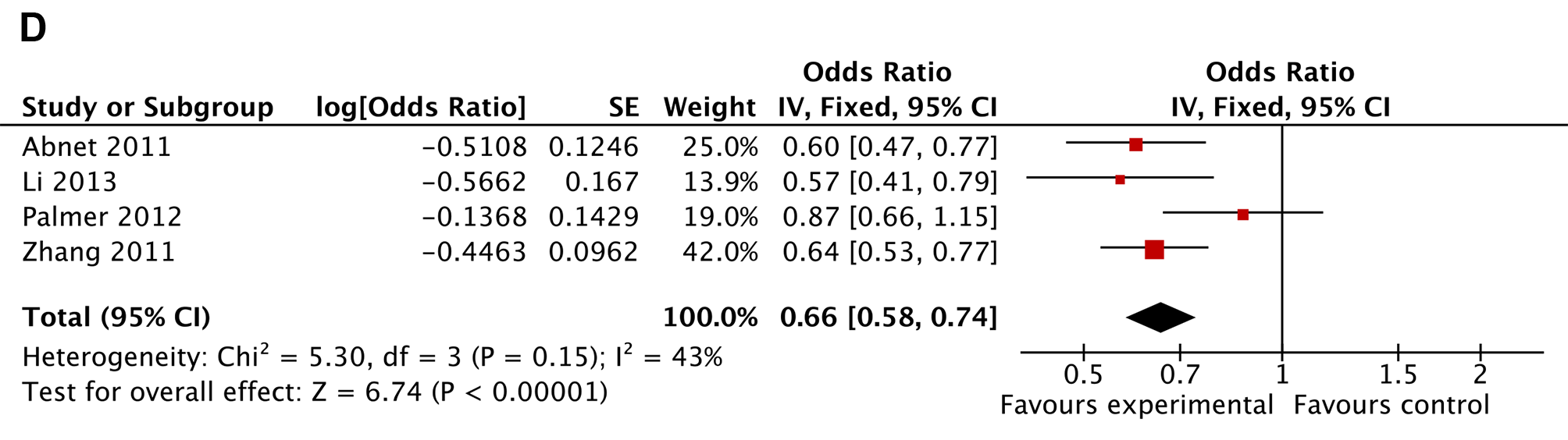


**Additional file 3. Forest plots describing subgroup analyses of *MUC1* rs4072037 and susceptibility to gastric cancer under the allele model.**

A. Intestinal-type gastric cancer; B. Diffuse-type gastric cancer; C. Cardia gastric cancer; D. Non-cardia gastric cancer.

The horizontal lines represent 95% CIs for estimating the outcome of the G allele versus the A allele in the meta-analysis.

(■) Overall estimates of the effects.

CI, confidence interval; OR, odds ratio.
